# Longitudinal Effects of Ketamine on Dendritic Architecture *In Vivo* in the Mouse Medial Frontal Cortex^1^,^2^,^3^

**DOI:** 10.1523/ENEURO.0133-15.2016

**Published:** 2016-04-04

**Authors:** Victoria Phoumthipphavong, Florent Barthas, Samantha Hassett, Alex C. Kwan

**Affiliations:** 1 Department of Psychiatry, Yale University, New Haven, Connecticut 06511; 2 Department of Neuroscience, Yale University, New Haven, Connecticut 06511

**Keywords:** dendrites, dendritic spines, frontal cortex, ketamine, structural plasticity, two-photon microscopy

## Abstract

A single subanesthetic dose of ketamine, an NMDA receptor antagonist, leads to fast-acting antidepressant effects. In rodent models, systemic ketamine is associated with higher dendritic spine density in the prefrontal cortex, reflecting structural remodeling that may underlie the behavioral changes. However, turnover of dendritic spines is a dynamic process *in vivo*, and the longitudinal effects of ketamine on structural plasticity remain unclear. The purpose of the current study is to use subcellular resolution optical imaging to determine the time course of dendritic alterations *in vivo* following systemic ketamine administration in mice. We used two-photon microscopy to visualize repeatedly the same set of dendritic branches in the mouse medial frontal cortex (MFC) before and after a single injection of ketamine or saline. Compared to controls, ketamine-injected mice had higher dendritic spine density in MFC for up to 2 weeks. This prolonged increase in spine density was driven by an elevated spine formation rate, and not by changes in the spine elimination rate. A fraction of the new spines following ketamine injection was persistent, which is indicative of functional synapses. In a few cases, we also observed retraction of distal apical tuft branches on the day immediately after ketamine administration. These results indicate that following systemic ketamine administration, certain dendritic inputs in MFC are removed immediately, while others are added gradually. These dynamic structural modifications are consistent with a model of ketamine action in which the net effect is a rebalancing of synaptic inputs received by frontal cortical neurons.

## Significance Statement

A single dose of ketamine leads to fast-acting antidepressant effects, and thus understanding its mechanism of action would facilitate the development of new treatments for mood disorders. One potential mechanism is the remodeling of synaptic connections, because ketamine administration in rodents leads to a higher density of dendritic spines in the frontal cortex. Structural remodeling, however, is a dynamic process, and the longitudinal effects of ketamine are poorly understood. In this study, we used subcellular resolution optical imaging methods to repeatedly visualize dendritic spines from the same set of neurons for >2 weeks in the mouse frontal cortex. The results are consistent with a model of action for ketamine involving the rebalancing of synaptic inputs in the frontal cortex.

## Introduction

Major depressive disorder is a top contributor to disease burden among mental illnesses in the United States (Murray et al., 2013). Core symptoms for depressive disorders are debilitating, yet treatment options are limited. Typical antidepressants require several weeks to months to be effective, and approximately one-third of patients remain nonresponsive even after multiple trials. In contrast to the slow onset of action for the currently available antidepressants, a single dose of ketamine produces antidepressant effects within several hours (Berman et al., 2000) and can last for up to 2 weeks (Ibrahim et al., 2012). Studies of ketamine effects in animal models have found antidepressant-like behavioral responses in naive and stressed rodents (Li et al., 2010; Autry et al., 2011; Li et al., 2011; Donahue et al., 2014). These studies have shed light on the molecular signaling pathways recruited by systemic ketamine administration. However, still unclear are the cellular and network mechanisms responsible for the behavioral improvements (Sanacora and Schatzberg, 2015).

One striking consequence of systemic ketamine administration in naive rodents is an increase in the dendritic spine density in the distal and proximal tufts of layer 5 pyramidal neurons in the medial prefrontal cortex (Li et al., 2010; Liu et al., 2013; Ruddy et al., 2015). These observations of synaptogenesis are in stark contrast with the structural and synaptic atrophy reported for patients with major depression (Drevets et al., 1997; Kang et al., 2012) and in chronic stress models (Cook and Wellman, 2004; Radley et al., 2004; Liston et al., 2006; Christoffel et al., 2011). The opposing effects of ketamine and stress on neural architecture suggest that there could be a structural basis for antidepressant actions. Namely, fast-acting antidepressants such as ketamine may restore synaptic connections that were lost in stress and mood disorders (Duman and Aghajanian, 2012). Indeed, when chronically stressed rats were injected with a single dose of ketamine, the stress-induced reduction in dendritic spine density could be reversed (Li et al., 2011).

However, the turnover of dendritic spines is a dynamic process *in vivo*. An increase in dendritic spine density could be due to an increase in formation rate, a decrease in elimination rate, or a combination of both factors. Moreover, newly formed spines can be transient or persistent, either disappearing or stabilizing after several days. It is unknown whether new spines following systemic ketamine are persistent and thus are associated with functional synapses (Knott et al., 2006). Characterizing these dynamics requires longitudinal methods. Two-photon microscopy is an optical imaging technique that enables the visualization of dendritic architecture *in vivo* at subcellular resolution for up to several months (Grutzendler et al., 2002; Holtmaat et al., 2009). This approach has been used to investigate structural plasticity following sensory experience (Trachtenberg et al., 2002), learning (Fu et al., 2012; Lai et al., 2013), and exposure to substances, including corticosterone (Liston and Gan, 2011) and cocaine (Muñoz-Cuevas et al., 2013).

In this study, we used two-photon imaging to characterize the effects of a single subanesthetic dose of ketamine on the dendritic architecture in the mouse medial frontal cortex (MFC). Our results showed that systemic ketamine leads to a relative increase in dendritic spine density, a prolonged change driven by an elevated rate of spine formation. A fraction of the ketamine-induced new spines was persistent and could be observed after 4 d, which is indicative of functional synapses. Unexpectedly, we also observed a loss of distal apical tuft branches that occurred specifically and immediately on the day after ketamine administration. These data demonstrate distinct short- and long-term consequences of ketamine on dendritic architecture, and highlight its impact on modifying the synaptic inputs impinging on frontal cortical neurons.

## Materials and Methods

### Mice

All animal procedures were performed in accordance with the regulations of the Yale University animal care committee. Experiments were performed on adult (postnatal day 73–149) *Thy1-GFP-M* (*n* = 13; #007788, The Jackson Laboratory, RRID:IMSR_JAX:007788) and *Thy1-YFP-H* transgenic mice (*n* = 3; #003782, The Jackson Laboratory, RRID:IMSR_JAX:003782). Mice of both sexes were used. Mice were housed under controlled temperature on a 12 h light/dark cycle with siblings (one to five per cage) and nesting material.

### Surgery

Anesthesia was induced with a 2% isoflurane and oxygen mixture, which was lowered to 1.5% for the remainder of the surgery. Mice were secured by ear bars in a stereotaxic frame. Their body temperature was regulated with a hot water circulation pad. Mice were injected with carprofen (5 mg/kg, s.c.; catalog #024751, Butler Schein Animal Health) and dexamethasone (40 mg/kg, i.m.; catalog #D4902, Sigma-Aldrich) prior to surgery. A 2- to 3-mm-diameter craniotomy was made over the right medial frontal cortex (AP = 1.5 mm, ML = 0.5 mm) with a handheld dental drill. After the skull was carefully removed, the surface of the brain was irrigated with an artificial cerebrospinal fluid (ACSF; in mm: 5 KCl, 5 HEPES, 135 NaCl, 1 MgCl2, and 1.8 CaCl2, pH 7.3) until bleeding subsides. A drop of warmed, low-melting point agarose solution (2% in ACSF; Type III-A, High EEO agarose, catalog #A9793, Sigma-Aldrich) was applied over the craniotomy. A two-layer glass plug was fabricated by first etching out a 2-mm-diameter circle from a #0 thickness glass coverslip, then bonded with UV-activated epoxy (NT37-322, Edmund Optics) to a #1 thickness, 3-mm-diameter round glass coverslip (catalog #64-0720-CS-3R, Warner Instruments). The glass plug was placed over the craniotomy and held in place until the agarose solidifies. The glass plug was then stabilized by applying light pressure and adding superglue around the edges. A stainless steel head plate was affixed to the skull using C&B-METABOND (Parkell Inc.). Mice were given another dose of carprofen (5 mg/kg, s.c.) immediately after surgery and for each of the following 3 d (5 mg/kg, i.p.). Mice were given a period of at least 3 weeks to recover before imaging begins.

### Imaging

Mice were anesthetized with 1.5% isoflurane and head fixed. Temperature was regulated using a heating pad with rectal probe feedback. The two-photon microscope (Movable Objective Microscope, Sutter Instrument) was controlled using the ScanImage software (Pologruto et al., 2003, RRID:SCR_014307). Excitation was provided by an ultrafast laser (Chameleon Ultra II, Coherent) and focused with a high-numerical aperture microscope objective (XLUMPLFLN20X/1.0, Olympus). For imaging green fluorescent protein (GFP)- or yellow fluorescent protein (YFP)-expressing dendrites, excitation wavelength was set at 920 nm, and emission was collected behind a bandpass filter from 475 to 550 nm. Each mouse was injected with either ketamine (10 mg/kg, i.p.) or saline vehicle on a non-imaging day. To investigate short-term effects, mice were imaged on days −3, −1, and 1 relative to the day of injection. For long-term studies, mice were imaged on days −3, −1, 1, 3, 5, 10, and 15 relative to the day of injection. Multiple fields of view were imaged in the same mouse. The same field of view was identified across days by finding landmark structures such as blood vessels or an edge of the glass window. At each field of view, image stacks were acquired at 1024 × 1024 pixels, spanning a field of view of 60.5 × 60.5 μm, and at 2 μm steps for a *z*-range of 20–30 μm. Each imaging session lasted up to 2.5 h. Although we did not explicitly record the duration of imaging sessions, we estimated *post hoc* based on the acquisition times of the first and last image files in the computer.

### Image analysis

In all of the figures, we are presenting the raw images with only adjustments to the black-and-white levels (linear), with no modification to contrast (nonlinear) or removal of neighboring axons, or any other manipulations. Initially, image stacks were processed for motion correction using the StackReg plug-in (Thévenaz et al., 1998, RRID:SCR_014308) in ImageJ (Schneider et al., 2012, RRID:SCR_003070). Then, structural parameters were analyzed from each image stack using ImageJ. The physical parameters of dendritic spines were characterized based on criteria established in a standardized protocol (Holtmaat et al., 2009). Briefly, dendritic spines were counted if the protrusions extended at least 0.4 μm away from the shaft. Dendritic spine length was the distance from the base at the shaft to the tip. Dendritic spine head diameter was the width at the widest extent of the spine. Distances were measured using the line segment tool in ImageJ. The dendritic spine formation rate was defined as the number of new spine protrusions observed in two consecutive imaging sessions divided by the total number of dendritic spines in the first imaging session. To assess longitudinal changes in the spine formation rate, we calculated the difference from baseline by subtracting the formation rate of each field of view by the baseline rate of the subject. The baseline rate of each subject was estimated by averaging the spine formation rates of all fields of view imaged from the same individual prior to injection (i.e. between days −3 and −1). The dendritic elimination rate was quantified using the same procedure for spine protrusions that disappeared. Most of the sessions were imaged 2 d apart, but some sessions were imaged 5 d apart (i.e., days 5–10 and days 10–15). Presumably, with the same spine formation rate, we would observe more new spines in sessions occurring 5 d apart relative to those occurring 2 d apart because more time has elapsed. Therefore, when estimating the spine formation/elimination rate from new/lost spine counts, we report turnover rates for sessions occurring 5 d apart with a correction factor, by multiplying the measured rates by two-fifths. For the apical tuft branches, dendritic segments were traced over using the freehand line tool, and then summed for total length in ImageJ. To assess longitudinal changes of the imaged dendritic segments, we calculated the fold change from the last session for each field of view by dividing the measured branch length of an imaging session by that of the prior imaging session.

### Statistics

We performed statistical tests considering fields of view as independent samples. This is a major assumption, justified in part by the fact that the fields of view were at random, nonoverlapping locations and that each one comprises a very small portion (0.06%) of the window area of each mouse. The reason for making this assumption is that a different number of fields of view was obtained for each mouse, so if we compare subjects only, the results will have a bias for those with fewer fields of view. To ensure that this assumption does not affect the major conclusions of the study, we repeated statistical tests in data from three sessions considering each mouse as a sample when possible. For all longitudinal results, two-way mixed ANOVA with repeated measures was used to test the factors contributing to changes in spine density, dendritic branch length, spine formation rate, and spine elimination rate. The factors were treatment (with ketamine or saline; between-subject), day (within-subject), and their interaction. The two-tailed *t* test was used to compare means that did not involve multiple days. The two-sample Kolmogorov–Smirnov test was used to compare cumulative distributions. Data are reported as the mean ± SEM. **Table 1** contains a list of the statistical tests performed, *p* values, and sample sizes. *p* values and sample sizes are reported instead of observed power to provide more information on the samples involved and because the *p* values are directly related to the observed power.

**Table 1:** Statistical table

		Data structure	Test	Exact *p* value	*N*
a	Spine density	Two-factor, btw (treatment) and win (day)	rANOVA	Treatment: *p* = 6 × 10^−7^; day: *p* = 0.40; interaction: *p* = 0.39	28/25 fields of view for 7 sessions for ket vs saline
b	Spine formation rate	Two-factor, btw (treatment) and win (day)	rANOVA	Treatment: *p* = 0.03; day: *p* = 0.001; interaction: *p* = 0.03	58/97 fields of view for 3 sessions for ket vs saline
c	Spine elimination rate	Two-factor, btw (treatment) and win (day)	rANOVA	Treatment: *p* = 0.9; day: *p* = 0.003; interaction: *p* = 0.9	58/97 fields of view for 3 sessions for ket vs saline
d	Spine formation rate	Two-factor, btw (treatment) and win (day)	rANOVA	Treatment: *p* = 2 × 10^−4^; day: *p* = 0.5; interaction: *p* = 0.08	28/25 fields of view for 7 sessions for ket vs saline
e	Spine elimination rate	Two-factor, btw (treatment) and win (day)	rANOVA	Treatment: *p* = 0.1; day: *p* = 0.001; interaction: *p* = 0.07	28/25 fields of view for 7 sessions for ket vs saline
f	Field of view fraction	Normally distributed	χ^2^ test	*p* = 0.005	58/97 fields of view for ket vs saline
g	Spine density	Two-factor, btw (treatment) and win (day)	rANOVA	Treatment: *p* = 0.007; day: *p* = 0.87; interaction: *p* = 0.98	8/8 mice for ket vs saline
h	Spine formation rate	Two-factor, btw (treatment) and win (day)	rANOVA	Treatment: *p* = 0.07; day: *p* = 0.20; interaction: *p* = 0.69	8/8 mice for ket vs saline
i	Spine elimination rate	Two-factor, btw (treatment) and win (day)	rANOVA	Treatment: *p* = 0.64; day: *p* = 0.23; interaction: *p* = 0.62	8/8 mice for ket vs saline
j	Persistent fraction	Normally distributed	Two-tailed *t* test	*p* = 0.3	28/25 fields of view for ket vs saline
k	Persistent fraction	Normally distributed	Two-tailed paired *t* test	*p* = 0.007	28 fields of view for ket
m	Persistent fraction	Normally distributed	Two-tailed paired *t* test	*p* = 0.002	28 fields of view for ket
n	Persistent fraction	Normally distributed	Two-tailed paired *t* test	*p* = 0.1	25 fields of view for saline
o	Persistent fraction	Normally distributed	Two-tailed paired *t* test	*p* = 0.9	25 fields of view for saline
p	Spine head length	Normally distributed	Two-tailed paired *t* test	*p* = 0.02	328/328 new vs existing spines
q	Spine head width	Normally distributed	Two-tailed paired *t* test	*p* = 3 × 10^−5^	328/328 new vs existing spines
r	Spine head length	Cumulative fractions	Two-sample Kolmogorov–Smirnov test	*p* = 9 × 10^−6^	328/328 new vs existing spines
s	Spine head width	Cumulative fractions	Two-sample Kolmogorov–Smirnov test	*p* = 4 × 10^−4^	328/328 new vs existing spines
t	Spine head length	Cumulative fractions	Two-sample Kolmogorov–Smirnov test	*p* = 0.9	61/328 spines for pre-ket vs post-ket
u	Spine head length	Cumulative fractions	Two-sample Kolmogorov–Smirnov test	*p* = 0.09	61/328 spines for pre-ket vs post-ket
v	Spine head width	Cumulative fractions	Two-sample Kolmogorov–Smirnov test	*p* = 0.2	61/328 spines for pre-ket vs post-ket
w	Spine head width	Cumulative fractions	Two-sample Kolmogorov–Smirnov test	*p* = 0.5	61/328 spines for pre-ket vs post-ket
x	Dendrite length	Two-factor, btw (treatment) and win (day)	rANOVA	Treatment: *p* = 1 × 10^−12^; day: *p* = 0.02; interaction: *p* = 0.02	28/25 fields of view for 7 sessions for ket vs saline
y	Dendrite length and formation rate	Two variables: binary (with or without branch loss) and continuous (formation rate)	Regression coefficient	*p* = 0.2	28 fields of view for ket
z	Dendrite length and elimination rate	Two variables: binary (with or without branch loss) and continuous (elimination rate)	Regression coefficient	*p* = 0.3	28 fields of view for ket
aa	Branch width of imaged dendritic segments	Normally distributed	Two-tailed *t* test	*p* = 0.44	117 stable and 16 retracted dendritic segments
ab	Dendrite length	Two-factor, btw (treatment) and win (day)	rANOVA	Treatment: *p* = 0.003; day: *p* = 0.69; interaction: *p* = 0.69	8/8 mice for ket vs saline
ac	Change in dendritic spine density	Non-parametric	Wilcoxon ranked-sum	*p* = 1	8/8 mice for ket vs saline
ad	Change in dendritic spine density	Non-parametric	Wilcoxon ranked-sum	*p* = 0.3	5 female and 11 male mice
ae	Change in dendritic spine density	Two continuous variables	Regression coefficient	*p* = 0.8	16 mice
af	Change in dendritic spine density	Two continuous variables	Regression coefficient	*p* = 0.8	16 mice
ag	Change in dendritic spine density	Two continuous variables	Regression coefficient	*p* = 0.16	16 mice

rANOVA, repeated measures ANOVA; btw, between-factor of the ANOVA; win, within-factor of the ANOVA; ket, ketamine administration; saline, saline administration.

## Results

### Longitudinal imaging of dendritic architecture in the mouse medial frontal cortex *in vivo*

**Figure 1.**
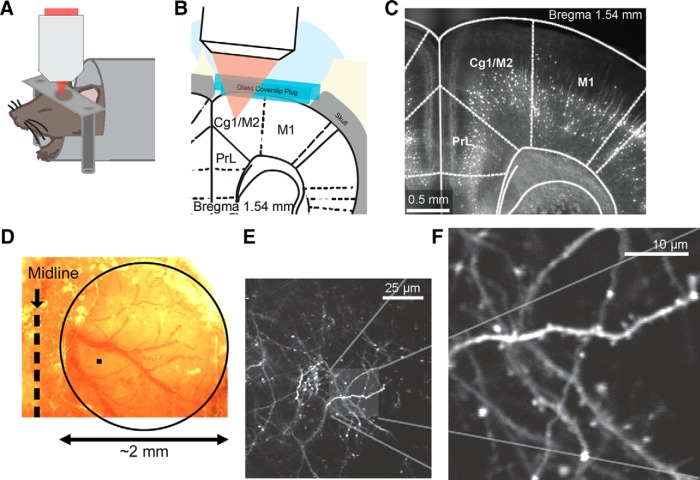
Longitudinal imaging of dendritic architecture in the mouse medial frontal cortex. ***A***, Schematic of the imaging experiment. ***B***, Schematic of the long-term window implant. ***C***, Fluorescence image of a fixed coronal brain slice from a *Thy1-GFP-M* mouse following longitudinal imaging. Cg1 and M2 (i.e., the MFC) were imaged in this study. PrL, prelimbic cortex. M1, primary motor cortex. ***D***, Bright-field image of the long-term window implant. The glass window has an ∼2-mm-diameter width (circle), which is much larger than the imaging field of view of ∼60 × 60 μm (filled square). ***E***, A low-magnification, *in vivo* two-photon image from layer 1 of the MFC in a *Thy1-GFP-M* mouse. Distal apical tuft branches from GFP-expressing layer 5 pyramidal neurons were visible. ***F***, A high-magnification image of a region in ***E***.

To visualize dendritic architecture, we performed two-photon microscopy (Fig. 1*A*,*B*) using the transgenic *Thy1-GFP-M* and *Thy1-YFP-H* mice (Feng et al., 2000), in which a sparse subset of neocortical neurons expresses the enhanced GFP or YFP. Many studies have used these mouse lines to investigate structural remodeling, but primarily in the sensory cortices (Trachtenberg et al., 2002; Knott et al., 2006). Therefore, we started by examining the distribution of fluorescent neurons in the frontal cortex. Fluorescence imaging of fixed coronal sections confirmed sparse labeling in anterior cingulate cortex (Cg1) and secondary motor cortex (M2; Fig. 1*C*). In these regions, fluorescence signals originated predominantly from layer 5 pyramidal neurons, as is evident from the laminar position of the cell bodies. This is consistent with the knowledge that only deep-layer pyramidal neurons are labeled in these two mouse lines (Feng et al., 2000). Interestingly, although there were no fluorescent cell bodies in the superficial layers, a band of the fluorescence signal could be seen in layer 2/3, particularly in the medial regions. This band may arise from axons in other brain regions, such as basolateral amygdala, that send long-range projections to frontal cortical regions (Oh et al., 2014).

In this study, we imaged layer 1 of the MFC, which includes Cg1 and M2. The choice of MFC was due to practical reasons because two-photon microscopy has depth limitations. Nevertheless, MFC is appropriate for studies of antidepressants as numerous studies have reported stress-induced structural and functional alterations in rodents, either specifically in the cingulate region (Liston et al., 2006; Ito et al., 2010; Kassem et al., 2013) or in a greater region that includes MFC (Radley et al., 2004; 2006; Cerqueira et al., 2007). These results are consistent with a recent brain-wide mapping study, which identified both Cg1 and M2 as regions with significantly reduced activity levels in a learned helplessness model of depression (Kim et al., 2016). Moreover, mapping of metabolic activity after systemic ketamine showed that MFC is among the activated brain regions in rodents (Duncan et al., 1999; Miyamoto et al., 2000). To prepare for longitudinal *in vivo* imaging, we performed a craniotomy above MFC and implanted a long-term ∼2-mm-diameter glass window (Fig. 1*D*). After recovery, mice were anesthetized with isoflurane and affixed on head posts under a two-photon microscope. Figure 1*E* shows a low-magnification image of the GFP-expressing dendrites in MFC *in vivo*. For counting dendritic spines, we acquired high-magnification 20- to 30-μm-thick image stacks at multiple fields of view (Fig. 1*F*). Individual dendritic branches could be distinguished from axons by the protruding dendritic spines along the segments. Because we were imaging superficial layers, these neuronal processes represented the distal apical tuft branches of layer 5 pyramidal neurons. We note that all images presented in this article have only linear adjustments to black-and-white levels, but have not otherwise been altered (see Materials and Methods).

### Systemic ketamine administration is associated with higher dendritic spine density in MFC for 2 weeks

**Figure 2.**
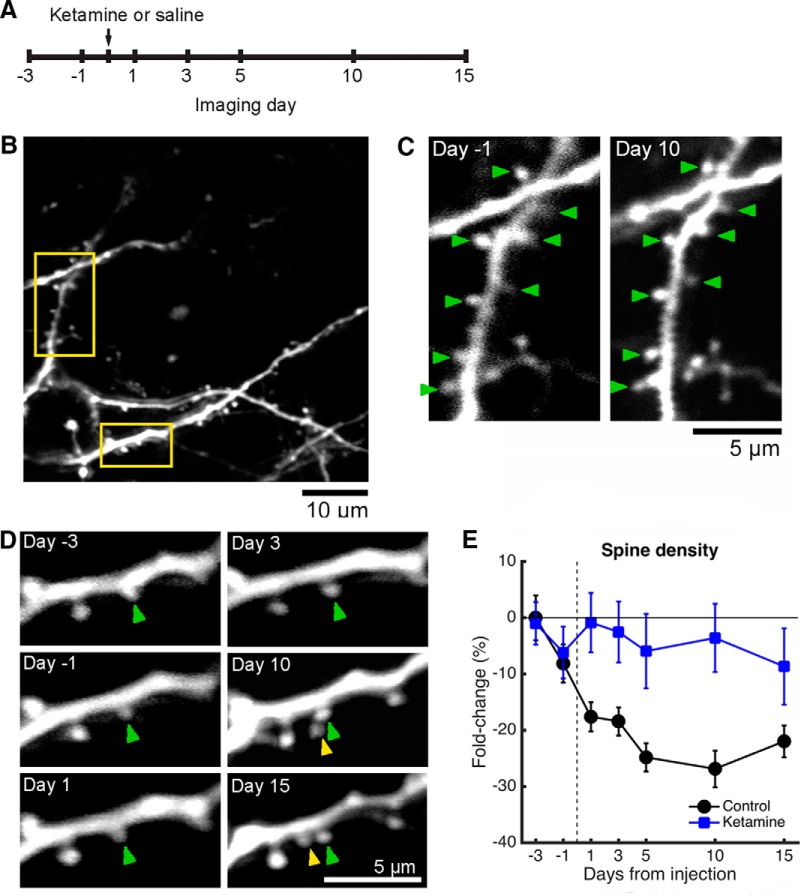
Systemic ketamine administration leads to higher dendritic spine density for at least 2 weeks relative to that of controls. ***A***, Time line of the experiment. Ketamine was administered at a dose of 10 mg/kg through intraperitoneal injection. ***B***, An example imaging field of view acquired on day −3 in a *Thy1-GFP-M* mouse. Yellow boxes indicate the dendritic branches shown as examples in ***C*** and ***D***. ***C***, Images of an apical dendritic tuft branch at days −1 and 10 from ketamine administration in a *Thy1-GFP-M* mouse. In the bottom right, axonal processes and boutons are visible. Green arrowhead, stable spine. ***D***, Another apical dendritic tuft branch from the same field of view at days −3, −1, 1, 3, 10, and 15 from ketamine administration in a *Thy1-GFP-M* mouse. A new spine (yellow arrowhead) appeared on day 10 next to a stable spine (green arrowhead). ***E***, Change in dendritic spine density across days, expressed as a fold change from the value measured on the first imaging session. The mouse was injected with either ketamine (blue square) or saline (black circle). Values are reported as the mean ± SEM. *N* = 28 and 25 fields of view across 7 sessions for ketamine- and saline-injected mice.

To examine the effects of ketamine on structural plasticity in the MFC, we visualized the same fields of view on multiple imaging sessions in adult mice, while administering either a single subanesthetic dose of ketamine (10 mg/kg, i.p.) or saline vehicle (Fig. 2*A*). We imaged on days −3, −1, 1, 3, 5, 10, and 15 from the injection day. We did not image on the injection day because anesthesia would interfere with neural activity, which is required for the antidepressant effects of ketamine (Fuchikami et al., 2015). We focused on the medial half of the 2-mm-diameter glass window. Image stacks were acquired from multiple, nonoverlapping fields of view (60.5 × 60.5 μm), each representing a tiny portion of the window area (0.06%; Fig. 1*D*). In total, we tracked 1,665 spines for ketamine (*n* = 8 mice; 58 fields of view, range 4–21 per mouse) and 3,814 spines for saline (*n* = 8 mice; 97 fields of view, range 4–17 per mouse). All the experiments involved at least the first three sessions. In a subset of experiments, we tracked dendritic architecture for the full seven-session period, including 800 spines for ketamine (*n* = 3 mice; 28 fields of view) and 783 spines for saline (*n* = 2 mice; 25 fields of view). For each field of view, we counted multiple branches including dozens of dendritic spines (mean = 38 spines/field of view, SD = 17). In the first imaging session, we measured the mean baseline dendritic spine density in MFC to be 0.28 spines/μm (SD = 0.08; *n* = 155 fields of view). This value for dendritic spine density is ∼25% lower than a previous measurement from the mouse dorsomedial prefrontal cortex (Muñoz-Cuevas et al., 2013), a difference that may be attributed to our mice being older adults.

Comparing the pre-ketamine administration and post-ketamine administration sessions, most dendritic spines were stable (Fig. 2*C*,*D*, green arrowheads). However, there were also instances where new spines were found (Fig. 2*D*, yellow arrowhead). To summarize data for ketamine and saline conditions, we quantified the fold change in dendritic spine density from baseline (day −3 from injection) for each field of view. Systemic ketamine was associated with higher dendritic spine density in the MFC (treatment: *p* = 6 × 10^−7^, *F*_(1,276)_ = 26.0; day: *p* = 0.40, *F*_(5,276)_ = 1.03; interaction: *p* = 0.39, *F*_(5,276)_ = 1.05; two-way ANOVA; Fig. 2*E*) relative to the saline group. It is noteworthy that we also observed a decline in dendritic spine density across days for saline-injected subjects (Fig. 2*E*, black line). This reduction of spine density in the “control” condition may be due to a number of factors, which will be discussed in a later section.

### Higher dendritic spine density is driven by an elevated rate of spine formation

**Figure 3.**
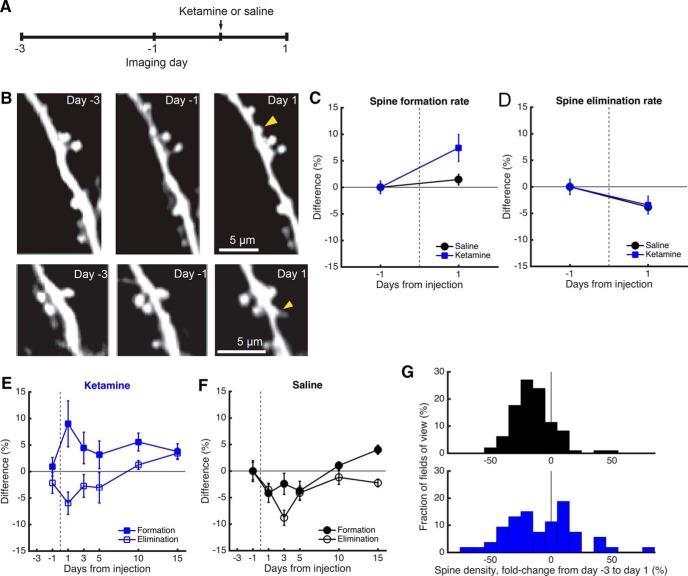
Higher spine density is due to an elevated rate of spine formation. ***A***, Time line of the experiment. Ketamine was administered at a dose of 10 mg/kg through intraperitoneal injection. ***B***, Images of two different apical dendritic tuft branches at days −3, −1, and 1 from ketamine administration in a *Thy1-GFP-M* mouse. Yellow arrowhead, new spine. ***C***, Change in spine formation rate, expressed as the difference from the value measured between days −3 and −1 (i.e., preinjection sessions). The mouse was injected with either ketamine (blue square) or saline (black circle). Values are reported as the mean ± SEM. *N* = 58 and 97 fields of view across three sessions for ketamine- and saline-injected mice. ***D***, Same as ***C*** for spine elimination rate. ***E***, Change in spine turnover dynamics across days for mice injected with ketamine. Solid square, spine formation rate. Open square, spine elimination rate. Values are reported as the mean ± SEM. ***F***, Same as ***E*** for controls with saline injection. *N* = 28 and 25 fields of view across seven sessions for ketamine- and saline-injected mice. ***G***, A histogram of the change in dendritic spine density, expressed as the fold change from day −3 to day 1 from injection. Top, saline. Bottom, ketamine. *N* = 58 and 97 fields of view for ketamine- and saline-injected mice.

Next we wanted to find the changes in dendritic spine turnover dynamics responsible for the relative increase in dendritic spine density. Because the largest spine density increase was found on the day after ketamine injection, we focused the analysis on the entire dataset across a period including days −3, 1, and 1 (Fig. 3*A*). Figure 3*B* shows two image montages of apical tuft branches before and after ketamine injection. To quantify spine turnover dynamics, we compared the same fields of view across consecutive imaging sessions to count the number of new and eliminated spines. Relative to the preinjection baseline, we found an increase in spine formation rate following systemic ketamine injection that was different from that found in the saline group (treatment: *p* = 0.03, *F*_(1,287)_ = 4.61; day: *p* = 0.001, *F*_(1,287)_ = 10.3; interaction: *p* = 0.03, *F*_(1,287)_ = 4.61; two-way ANOVA; Fig. 3*C*). In contrast, although there were changes in spine elimination rates across days, there was no difference between mice that received ketamine or saline (treatment: *p* = 0.9, *F*_(1,286)_ = 0.02; day: *p* = 0.003, *F*_(1,286)_ = 9.09; interaction: *p* = 0.9, *F*_(1,286)_ = 0.02; two-way ANOVA; Fig. 3*D*). We also plotted the spine turnover rates using only the seven-session dataset for the ketamine (Fig. 3*E*) and saline groups (Fig. 3*F*). Ketamine remained a significant factor contributing to a difference in spine formation rate (treatment: *p* = 2 × 10^−4^, *F*_(1,267)_ = 14.5; day: *p* = 0.5, *F*_(5,267)_ = 0.89; interaction: *p* = 0.08, *F*_(5,267)_ = 1.96; two-way ANOVA), but not in the spine elimination rate (treatment: *p* = 0.1, *F*_(1,267)_ = 2.79; day: *p* = 0.001, *F*_(5,267)_ = 4.19; interaction: *p* = 0.07, *F*_(5,267)_ = 2.03; two-way ANOVA). These results indicate that an elevated rate of spine formation is the driving force behind the higher spine density in the MFC following ketamine administration.

Although the mean spine formation rate was higher for mice with systemic ketamine administration relative to saline administration, there was variability across fields of view (Fig. 3*G*). As described previously, there was a decline in spine density across days in saline-injected subjects, and accordingly 83% of the imaged field of dendritic tuft branches had reduced spine density compared with the first-day baseline. By contrast, about half of the fields of view had an increase in spine density following ketamine injection (40%; *p* = 0.005, χ^2^ = 7.8, χ^2^ test). Using fields of view allows us to examine more finely the variability in the data; however, the results could be correlated among fields of view from the same individual. Therefore, we verified, on a per-subject basis across 7 d, that there is a significant effect of treatment on dendritic spine density (treatment: *p* = 0.007, *F*_(1,16)_ = 9.39; day: *p* = 0.87, *F*_(5,16)_ = 0.35; interaction: *p* = 0.46, *F*_(5,16)_ = 0.98; two-way ANOVA), that there is an effect that was near to but did not reach statistical significance for the effect of treatment on spine formation rate (treatment: *p* = 0.07, *F*_(1,16)_ = 3.87; day: *p* = 0.96, *F*_(5,16)_ = 0.20; interaction: *p* = 0.69, *F*_(5,16)_ = 0.62; two-way ANOVA), and that there is no effect of treatment on spine elimination rate (treatment: *p* = 0.64, *F*_(1,16)_ = 0.23; day: *p* = 0.23, *F*_(5,16)_ = 1.56; interaction: *p* = 0.62, *F*_(5,16)_ = 0.71; two-way ANOVA).

### A fraction of the newly formed spines associated with ketamine administration is persistent

**Figure 4.**
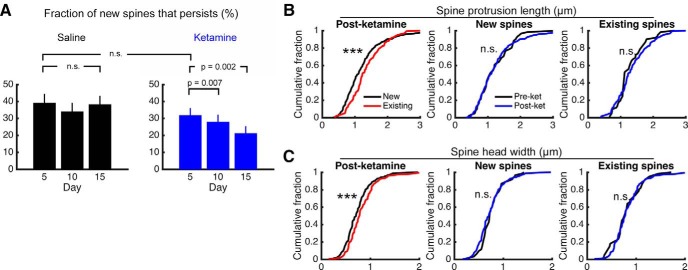
Newly formed protrusions following systemic ketamine administration are consistent with nascent spines. ***A***, The fraction of newly formed spines found on day 1 that could be observed again on day 5, 10, or 15 for mice injected with saline (black) or ketamine (blue). Paired *t* test for comparisons across days in the same condition. Unpaired *t* test for the comparison across conditions. The *p* values are shown as is without multiple-comparison correction. Values are reported as the mean ± SEM. *N* = 28 and 25 fields of view for ketamine- and saline-injected mice. ***B***, Distribution of spine protrusion lengths, comparing newly formed spines and existing stable spines that were on the same dendritic branch. Measurements were taken either before ketamine administration, on day −1, or after ketamine administration, on day 1, 3, 5, 10, or 15. ***, *p* < 0.001, two-sample Kolmogorov–Smirnov test. *N* = 61 new spines and 61 matched existing neighboring spines measured before ketamine administration. *N* = 328 new spines and 328 matched existing neighboring spines measured after ketamine administration. ***C***, Same as ***B*** for spine head widths.

An important question is whether the new dendritic spines associated with ketamine administration become functional synapses. A previous study (Knott et al., 2006) correlated images from two-photon and electron microscopy to show that a fraction of the newly formed dendritic spines is transient and disappears, whereas persistent spines that are stable for >4 d had synapses. For the new spines that were observed on the day following ketamine or saline injection, we quantified the fraction that could be observed at the same location 4 d later. Across fields of view, we found no difference in the fraction of spines that became persistent for ketamine versus that for saline (mean ± SEM: ketamine, 39 ± 5%; saline, 32 ± 4%; *p* = 0.3, *t*_(40)_ = 0.99, unpaired *t* test; Fig. 4*A*). However, the persistent fraction decreased over longer periods for ketamine-injected mice (day 5 vs day 10: *p* = 0.007, *t*_(17)_ = 3.08; day 5 vs day 15: *p* = 0.002, *t*_(15)_ = 3.85; paired *t* test, exact *p* values were reported without multiple-comparison adjustment), whereas it was unchanged for saline-injected mice (day 5 vs day 10: *p* = 0.1, *t*_(19)_ = 1.74; day 5 vs day 15: *p* = 0.9, *t*_(12)_ = −0.17; paired *t* test, exact *p* values were reported without multiple-comparison adjustment).

Furthermore, larger spines are known to correlate with more mature and stronger synaptic connections (Kasai et al., 2003). We measured the length and width of spine heads, comparing between newly formed spines, and matched each of those with a neighboring stable spine on the same dendritic branch. Relative to existing spines, new spines that appeared immediately on the day following systemic ketamine were shorter (mean ± SEM: new, 1.25 ± 0.04 μm, *n* = 328; existing, 1.35 ± 0.03 μm, *n* = 328; *p* = 0.02, *t*_(327)_ = −2.39, paired *t* test) and narrower (mean ± SEM: new, 0.74 ± 0.01 μm; existing, 0.83 ± 0.02 μm; *p* = 3 × 10^−5^, *t*_(327)_ = −4.19, paired *t* test). These differences in averages were reflected as differences in the cumulative distributions as well (spine length: *p* = 9 × 10^−6^, *D*_(328,328)_ = 0.19; spine width: *p* = 4 × 10^−4^, *D*_(328,328)_ = 0.16; two-sample Kolmogorov–Smirnov test; Fig. 4*B*,*C*). However, when we compared pre-ketamine administration with post-ketamine administration conditions, we did not find any difference in dendritic spine morphology (spine length, new spines: *p* = 0.9, *D*_(61,328)_ = 0.08; spine length, existing spines: *p* = 0.09, *D*_(61,328)_ = 0.17; spine width, new spines: *p* = 0.2, *D*_(61,328)_ = 0.15; spine width, existing spines: *p* = 0.5, *D*_(61,328)_ = 0.12; two-sample Kolmogorov–Smirnov test). The distributions of spine protrusion length and spine head width did not suggest obvious ways to segment the data, and therefore we did not attempt to identify types (i.e., stubby, mushroom like, or filopodia like). Together, these results indicate that newly formed protrusions following systemic ketamine administration have similar morphological characteristics to those that occurred before systemic ketamine administration. The new spine heads are shorter and narrower, which is broadly consistent with nascent spines that precede synapse formation. Nevertheless, a fraction of these spines that formed after systemic ketamine administration becomes persistent and likely reflects new synaptic connections.

### Ketamine also leads to rapid retraction of distal apical tuft branches

**Figure 5.**
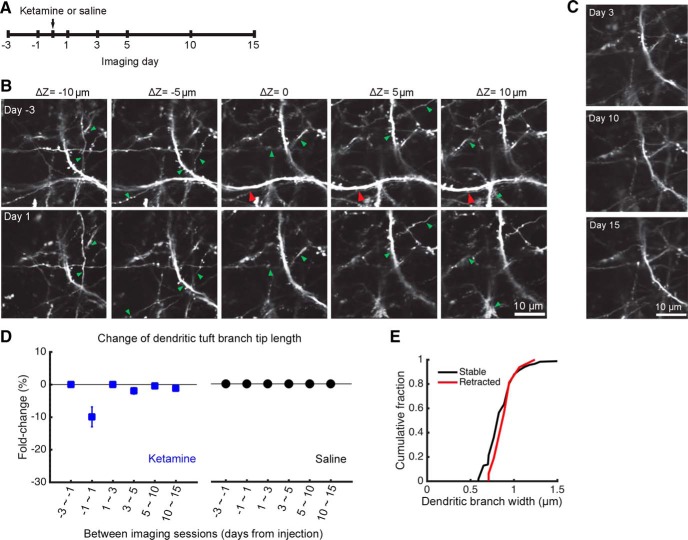
Systemic ketamine administration associated with the retraction of distal apical tuft branches. ***A***, Time line of the experiment. Ketamine was administered at a dose of 10 mg/kg through intraperitoneal injection. ***B***, Images from multiple *z*-depths of a volumetric acquisition of dendritic architecture obtained in a *Thy1-GFP-M* mouse before and after ketamine administration. Note that, although most branch segments were stable (green arrowhead), a segment in the middle of the volume has retracted (red arrowhead). ***C***, Same field of view as ***B*** at days 3, 10, and 15 from ketamine administration. ***D***, Change in distal apical tuft branch length in layer 1 across days, with the fold change calculated by dividing the length of each session by that from the prior session. The mouse was injected with either ketamine (blue square) or saline vehicle (black circles). Values are reported as the mean ± SEM. *N* = 28 and 25 fields of view across seven sessions for ketamine- and saline-injected mice. ***E***, Distributions of dendritic branch widths measured on day −1, plotted separately for those distal apical tuft branches that were stable (black) or retracted (red) on day 1. *N* = 117 stable and 16 retracted dendritic segments from ketamine-injected mice.

Unexpectedly, we also observed alterations to the distal apical tuft branches following ketamine injection in a fraction (18%) of the fields of view (Fig. 5*A*). Figure 5*B* shows the same field of view across imaging sessions where a distal branch segment was visible in a preinjection session (Fig. 5*B*, red arrowheads), and then disappeared on the day following systemic ketamine administration. This observation was not due to out of focus imaging, because we acquired volumetric image stacks where neuronal processes below and above the image plane were clearly visible and stable (Fig. 5*B*, green arrowheads). No additional alterations were observed in the subsequent days (Fig. 5*C*). Analysis of the longitudinal dataset revealed a mean change of −10 ± 3% in the total length of the imaged apical tuft branches on day 1 after ketamine injection (treatment: *p* = 1 × 10^−12^, *F*_(1,236)_ = 56.5; day: *p* = 0.02, *F*_(5,236)_ = 2.77; interaction: *p* = 0.02, *F*_(5,236)_ = 2.77; two-way ANOVA; Fig. 5*D*). We compared fields of view with and without apical tuft branch loss and found no differences in their spine turnover rates (*p* = 0.2, formation; *p* = 0.3 elimination; unpaired *t* test). We also asked whether stable and retracted dendritic branches had different widths, but did not find any difference (*p* = 0.4, *t*_(131)_ = −0.77, unpaired *t* test; Fig. 5*E*). Additional statistical tests on a per-subject basis for the three-session data confirmed a significant effect of treatment on apical tuft branch length (treatment: *p* = 0.003, *F*_(1,16)_ = 12.08; day: *p* = 0.7, *F*_(5,16)_ = 0.62; interaction: *p* = 0.7, *F*_(5,16)_ = 0.62; two-way ANOVA). These results show that systemic ketamine has a short-term effect of removing a small portion of the apical dendritic tuft branches in layer 1.

### Potential factors contributing to the decline of dendritic spine density prior to injection

**Figure 6.**
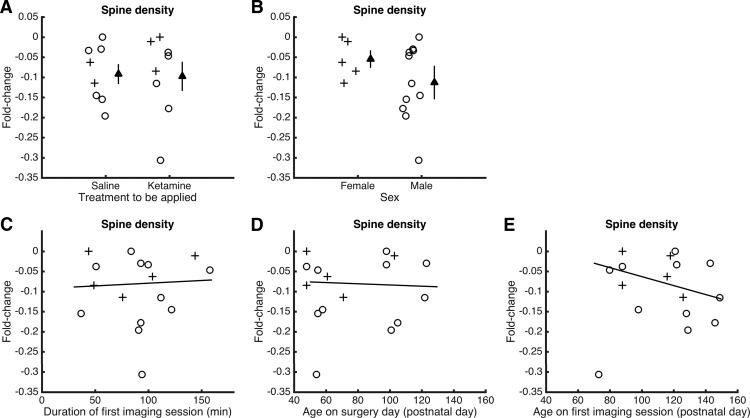
Potential factors contributing to the decline of dendritic spine density prior to injection. ***A***, Fold change in dendritic spine density from day −3 to day −1 (preinjection) for mice to be injected with saline or ketamine. Circle, male. Cross, female. Filled triangle, mean ± SEM. ***B***, Same as ***A*** for female vs male mice. ***C***, Fold change in dendritic spine density from day −3 to day −1 (preinjection) plotted as a function of the duration of the imaging session on day −3. Circle, male. Cross, female. Line, linear fit excluding the outlier at −0.3. ***D***, Same as ***C*** for age at the time of surgery. ***E***, Same as ***C*** for age at the time of the first imaging session.

We observed a decline in dendritic spine density in saline-injected mice. To further investigate the potential contributing factors, we examined changes in spine density during the preinjection period, between days −3 and −1. There were no significant differences between mice to be injected with saline versus those to be injected with ketamine (*p* = 1, Wilcoxon rank-sum test; Fig. 6*A*), or between male and female subjects (*p* = 0.3, Wilcoxon rank-sum test; Fig. 6*B*). We conjecture that stress could arise from the duration of anesthesia required for imaging, but found no systematic trend between imaging session duration and changes in spine density (*p* = 0.8; *t*_(13)_ = 0.26, linear regression, excluding an outlier at −0.3; Fig. 6*C*). There was also no significant trend for the age of the animal at the time of glass window implantation (*p* = 0.8; *t*_(13)_ = −0.23, linear regression, excluding outlier at −0.3; Fig. 6*D*). A potential contributor is the age of the animal at the time of imaging, where older adults tended to have larger declines in dendritic spine density (*p* = 0.16; *t*_(13)_ = −1.50, linear regression, excluding an outlier at −0.3; Fig. 6*E*), although this effect did not reach significance. We should note that there have been a couple of other reports (Wellman, 2001; Muñoz-Cuevas et al., 2013) of structural loss in rodent prefrontal cortex in control or vehicle-injected animals. These earlier studies along with our own data highlight the difficulty in achieving true controls in studies of frontal cortex, where the brain region is known to be sensitive to aversive life events.

## Discussion

Our time-lapse results demonstrated higher dendritic spine density for up to 2 weeks after a single dose of ketamine relative to saline. This is a consequence of an elevated rate of spine formation. We also observed a loss of distal apical tuft branches that was specific to the day following ketamine injection. The short- and long-term effects on apical tuft branches and dendritic spine density would have opposite effects on the overall number of synaptic connections. By removing certain inputs immediately and adding others gradually, we suggest that ketamine may act to reorganize the types of synaptic inputs received by pyramidal neurons in the MFC. Physiological evidence hinted at this possibility; in the frontal cortex, hypocretin-sensitive synaptic inputs, likely distinct from those that mediate serotonergic signaling, originate from the thalamus. Intriguingly, although ketamine restores the magnitude of these synaptic currents in stressed rats, they appear to reach different levels relative to baseline (Li et al., 2011). Further experiments are needed to confirm the identities of the added and lost synaptic connections following systemic ketamine administration.

Our study builds on previous studies (Li et al., 2010, 2011) of ketamine in naive rats and chronic stress models, which found an increase in dendritic spine density in the distal and proximal tufts of layer 5 pyramidal neurons. These studies examined structural changes in the anterior cingulate and prelimbic regions by filling cells in brain slices prepared 24 h after treatment. Here, investigating effects *in vivo*, we found a relative increase in spine density in the MFC, which is more dorsally located but still part of the rodent medial prefrontal cortical network (Van De Werd et al., 2010; Vogt and Paxinos, 2014). We should emphasize that the observed relative increase is a result of dendritic spine density remaining mostly stable for ketamine, but declining for saline-injected mice. The decline of spine density in the saline group suggests that mice might have been stressed inadvertently in our experiments, potentially as a function of age at the time of imaging. Interestingly, other studies have observed an increase in dendritic spine density following a single dose of another rapid-acting antidepressant, scopolamine (Voleti et al., 2013), and reversal of stress-induced atrophy by long-term administration of fluoxetine (Bessa et al., 2009). Therefore, our results and studies in the field (Bessa et al., 2009; Li et al., 2010, 2011; Voleti et al., 2013) support a structural basis for antidepressant actions that may generalize beyond specific frontal cortical regions or pharmacological agents.

A novel finding is that the higher dendritic spine density after systemic ketamine administration is due to an elevated spine formation rate, but not to changes in the spine elimination rate. This increase in spine formation rate was largest on the day after systemic ketamine administration. For the later imaging sessions, the spine formation rate remained above the baseline, preinjection levels. This time course of elevated spine formation rate may be compared with the time course of the antidepressant effects of systemic ketamine. In rats, depressive-like behaviors, as assayed by forced swim and sucrose preference tests, were reduced 1 week after the injection of ketamine (Autry et al., 2011; Li et al., 2011). In patients with major depressive disorder, the duration of the antidepressant effects of ketamine varies from 3 d to 2 weeks (Ibrahim et al., 2012). Therefore, the long-term effect on dendritic spine turnover may relate to the sustained antidepressant effects observed in rodents and humans. Furthermore, the observation of changes in the dynamics of the spine formation rate, but not in other structural plasticity parameters, suggests that antidepressant effects may rely on molecular pathways that promote synaptogenesis, rather than those related to spine growth or pruning.

Several factors may influence the rates of ketamine-induced structural remodeling. Sex is a contributing variable because estrogen is known to affect structural plasticity (Srivastava et al., 2008). In this study, sex differences were not tested explicitly, owing to the limited sample size. Two lines of evidence suggest that pooling the data from males and females should not affect the conclusions of this study. First, we repeated the analysis using data from the five ketamine-injected males only, and found similar trends for ketamine-induced changes, including a relative increase in dendritic spine density and an elevated spine formation rate, but no change in the spine elimination rate. Second, two recent studies (Carrier and Kabbaj, 2013; Franceschelli et al., 2015) reported that although female rats are more sensitive to the antidepressant-like effects of ketamine at low dose, behavioral outcomes are similar between males and females at higher doses. Here, we used a dose (10 mg/kg) at which these studies found comparable behavioral effects for the sexes. Another potential variable is the surgical method. One report (Xu et al., 2007) argued that open-skull craniotomy can alter dendritic spine turnover rates, although another study (Holtmaat et al., 2009) found negligible differences across surgical preparations. The same procedures were applied to the ketamine and saline groups in our study; therefore, the influence of surgical methods on the across-group differences should be minimal. Furthermore, ketamine is often used with xylazine as an anesthetic. There is evidence that an anesthetic dose of ketamine has transient effects on the dynamics of dendritic filopodia but no effect on dendritic spines in 1-month-old mice (Yang et al., 2011). It is unclear how this prior result compares with the current findings, because we used a subanesthetic dose.

A surprising observation was the retraction of distal apical tuft branches, specific to the day after systemic ketamine administration. We emphasize that the imaged branch segments reside in the superficial layers of the cortex, therefore representing the distal portion and only a tiny fraction of the entire dendritic tree of a neuron. The loss of apical tuft branch tips may be due to the retraction of dendritic branches or the death of the neurons from which these dendrites arise, possibilities that could not be distinguished from our data. Nevertheless, our result was unexpected because, although some cortical cell types such as GABAergic interneurons can undergo branch tip reorganization under certain conditions (Chen et al., 2011), the dendritic branches of pyramidal neurons are thought to be stable in the neocortex of adult mice (Grutzendler et al., 2002). Studies have shown that structural plasticity in MFC is important for cognitive behaviors such as consolidation of contextual memory (Vetere et al., 2011) and adaptive decision-making (Liston et al., 2006; Dias-Ferreira et al., 2009). Therefore, the loss of dendritic materials may contribute to cognitive impairments, which are known to affect long-term ketamine users (Morgan et al., 2009). At higher dosages, repeated ketamine use has been associated with a reduced volume of hippocampus and frontal lobe in humans (Liao et al., 2011) and rodents (Kassem et al., 2013; Schobel et al., 2013). One correlated functional imaging study (Kassem et al., 2013) showed that such gray matter reduction is primarily due to a loss of dendrites and their synapses. There are ongoing efforts in the field to develop compounds with ketamine-like antidepressant actions but without the psychotomimetic effects, and it would be interesting to test whether those drugs may promote structural plasticity but spare dendritic material loss.

The short- and long-term effects of distal tuft branch loss and elevated spine formation rate have opposing effects on the total number of dendritic spines in the MFC. Long-range inputs into the superficial layers of rodent MFC come from multiple sources, including mediodorsal and midline thalamic nuclei, basolateral amygdala, and other prefrontal cortical areas (Hoover and Vertes, 2007; Oh et al., 2014). Specific types of prefrontal cortical inputs and outputs may be more plastic and susceptible to stress or ketamine (Shansky et al., 2009; Liu et al., 2015). Therefore, approaches that can alter prefrontal cortical circuitry with pathway specificity may be effective treatment options for mood disorders and merit further study.

## Synthesis

The decision was a result of the Reviewing Editor Christophe Bernard and the peer reviewers coming together and discussing their recommendations until a consensus was reached. A fact-based synthesis statement explaining their decision and outlining what is needed to prepare a revision is listed below. The following reviewers agreed to reveal their identity: Erik Bloss, Juan Nácher

Synthesis of reviews for:

eN-NWR-0133-15 "Longitudinal effects of ketamine on dendritic architecture in vivo in the mouse medial frontal cortex"

Please note that all the reviewers' comments have been integrated to reflect their collective impressions, queries, and suggestions for additional experiments and/or analysis and revisions:

I. Summary

The present manuscript investigates the effects of a single sub anesthetic dose of ketamine on the structural plasticity of superficial dendritic segments of pyramidal neurons in the frontal cortex. The authors study changes in dendritic spine density and dynamics as well as assessing the length of distal apical tuft branches by repeated 2 photon imaging in glass- windowed, transgenically labeled mice. The main finding is that ketamine treatment differentially changes spine dynamics, transiently increasing the rate of spine formation but not spine elimination. Additionally, the authors show some transient remodeling of distal apical tuft dendrites in ketamine but not saline treated mice.

Previous studies have described increases in spine density in pyramidal neurons after ketamine administration. This study extends prior work in that the authors were able to follow the same dendritic fragments over several weeks using 2 photon microscopy and by their findings that the net increase in spine density subsequent to a single dose of ketamine arises from an elevated spine formation rate without alteration in the rate of disappearance of the spines. The authors also find an interesting fast retraction of distal apical tuft branches.

The reviewers agreed that the study was interesting and added to our knowledge of the neurobiological bases of the fast antidepressant actions of NMDA receptor antagonists.

The reviewers also agreed, however, that the findings are less compelling due to two serious potential confounds and the lack of inclusion of more detailed analysis of the branch retraction effect. The first confound is that all of measures are made against a substantial overall loss of spines (as revealed by examination of the saline treated mice). The second confound is the inclusion of (and mixing of data from) both female and male mice. In light of the strong effects that estrogens have on structural plasticity and particularly on spine dynamics, pooling males and females makes the comparison problematic, especially with the low n values employed here. Although the suggested examination of effect by sex might resolve this issue, the phase of the estrous cycle in which the female mice were subjected to analyses should also be considered. Lastly, the authors need to provide some more data on the wholesale retraction of branches. Since the imaging they are doing is relatively straightforward (i.e., in an anesthetized mouse with LV neurons labeled by transgene), the branch retraction effect should be examined in greater detail, and potentially extended with data on axonal structural plasticity (which they should have already).

In summary: the major issues that must be addressed by the authors before the manuscript is suitable for publication in eNeuro are:

1. Examine the statistics by sex to assess the potential effect of the inclusion of females. (does this explain the particularly high degree of baseline spine retraction, perhaps?)
2. Include or obtain new data on the spine loss over time in non-injected animals (does the stress of injection per se account for the elevated baseline of retraction?) and
3. provide some way in which we can understand the high baseline retraction. Whether this means new data or new analysis it certainly should be discussed in more detail.

II Specific Issues: Major

The pooling of male and female mice is problematic given the known fluctuations of spine density in relation to the estrous cycle in female mice, rats, and non-human primates. It would be useful to consider each dataset by sex rather than pooling them together. In fact, given the report by Srivastava et al (PMID:18801922) of two-stage, estrogen+NMDA receptor dependent rewiring, sex differences as a result of ketamine treatment could be interesting. As the n is already low in the ketamine group (n=4), new animals might need to be imaged.

The strongest effect reported in the paper is the spine density decrease over time in the saline treated mice. Compared to the decrease seen by Munoz-Cuevas et al under similar conditions/brain region, the relative spine density change reported here is substantially larger over the same time span (e.g., ∼30% reduction after 11 days in the present work compared to only ∼5% in the Munoz-Cuevas et al report). Previous work from Yang et al (PMID:21768874) would argue against large effects of anesthesia on spines. Although the authors have devoted some text to this finding, can they provide a more realistic rationale as to why spines are being lost at such a high rate in their control preparation? Is there data from non-injected control mice that also show this effect?

A similar fraction of newly formed spines appear to be persistent (at 4 days) between the ketamine and saline groups. Can the authors look at a longer timescale? If not this section of the manuscript should minimized, as it brings little to the table.

The wholesale retraction of dendrites over a small time period is surprising. In particular, the example from Fig. 5 appears to be a thick tuft branch, implying that whole sets of branch families could be remodeled. If this is the case, how do the authors arrive at a 10% change without any knowledge of the dendritic arbor morphology? Are the branches that are lost generally thick, thin, or both? Is it possible to use large field of view imaging to document this effect within the context of a more complete arbor?

III Specific Issues: Minor

1. The authors presumably have both longitudinal series of cortical and subcortical axons in their acquired datasets. It would be useful to know how bouton dynamics are affected by ketamine, and whether they form dynamic or static subpopulations.
2. The paragraph on page 16 ("A novel finding...") needs some focus. It was hard to understand exactly what the authors were getting at.
3. One of the reviewers takes issue with the sentence in the significant statement "Ketamine administration in rodents leads to more synaptic connections in the frontal cortex, measured as higher density of dendritic spines." An increase in synaptic connections does not necessarily mean an increase in dendritic spines, since new synaptic contacts could be made on preexisting spines or in the dendritic shaft. Moreover, an increase in dendritic spines does not necessarily mean an increase in synaptic connections, because the proportion of the new spines might not receive synaptic inputs.
4. It would be helpful for the reader to have a sentence stating that the strain of mice used in this study only has labeled pyramidal neurons in the frontal cortex. This is important because other similar strains (e.g. Thy1-S mice) also display fluorescent interneurons.
5. The authors should give more information on the age of the animals when they started the experiments. It is possible that significant differences in the structural plasticity exist between 2 month old and 4 month old animals.
6. Please include reference for Image J software (http://rsbweb.nih.gov/ij/docs/faqs.html#cite)
7. It would be interesting to know whether there are differences in the density of the type of spines (stubby, mushroom, filopodia-like...) detected before and after ketamine treatment. Or at least have a sentence in the discussion stating why these type of analysis cannot be performed in this experiment.
8. The authors should make clearer that the increases in spine density induced by ketamine are detected when comparing the treatment with the effect of saline. This part of the results is a bit confusing, because if one only follows the effect of ketamine, it induces a decrease in this parameter (or virtually no change at 1 day post injection.
9. I think that the authors should also consider the possibility that the disappearance of some terminal tips of dendrites in layer could be due to the death of certain neuronal populations from which these dendrites arise. They also should cite the work by Nedivi's lab, who found similar dendritic tip remodeling in control cortical interneurons.
10. In page 16 the authors should provide references for their statement: "Therefore, our results and studies in the field support a structural basis for antidepressant actions that may generalize beyond specific frontal cortical regions or pharmacological agents".
11. I do not know whether this is possible, because the authors do not give information on whether animals used in the study were perfused at the end. But if this were the case, it would be extremely interesting to investigate what is the main input onto the dendrites/spine under analysis. A simple analysis with VGLUT1 and VGLUT2 receptors would determine the extra/intracortical origin of the projections.
